# SARS-CoV-2-Cholesterol Interaction: A Lot of Food for Thought

**DOI:** 10.3390/pathogens10010032

**Published:** 2021-01-03

**Authors:** Giovanni Di Guardo

**Affiliations:** Faculty of Veterinary Medicine, University of Teramo, Localita’ Piano d’Accio, 64100 Teramo, Italy; gdiguardo@unite.it

With over 80 million cases, and 1,800,000 deaths reported at the end of 2020 by the World Health Organization, the “CoronaVirus Disease-2019” (CoViD-19) pandemic, caused by the Severe Acute Respiratory Syndrome Coronavirus 2 (SARS-CoV-2), should be viewed as a global catastrophe. 

In the only 12 months hitherto elapsed from SARS-CoV-2 identification and genomic characterization [1], while safe, effective, and officially approved anti-CoViD-19 vaccines are now available to be progressively administered to mankind worldwide, an unprecedented and incredible amount of data have also been published about the SARS-CoV-2 infection pathogenesis and virus–host relationships.

Within this challenging and intriguing context, the selective interaction taking place between the viral spike (S) protein’s subunit 1 (S1) and the high-density lipoprotein (HDL) scavenger receptor B type 1 (SR-B1), which has been elegantly described in a recent article by Dr Wei and coworkers [2], significantly expands our knowledge on host–pathogen relationships. More in detail, following SARS-CoV-2 interaction with HDL-bound cholesterol and, thereafter, with the SR-B1 molecule expressed by pulmonary tissues, as well as by a variety of extra-pulmonary tissues, a facilitated viral entry into angiotensin-converting enzyme-2 (ACE-2)-harbouring cells is observed [2]. 

This is of interest when considering the widespread distribution of the ACE-2 viral receptor throughout host cells and tissues [3], on one side, along with SR-B1 co-expression by several of the aforementioned cytotypes [2], on the other. Within such an intricate landscape, adequate attention should be also paid to the pivotal role played by transmembrane serine protease 2 (TMPRSS2), a furin-like enzyme acting on the viral S1 receptor binding domain (RBD) polybasic cleavage site, thus enabling SARS-CoV-2 RBD attachment to ACE-2-expressing cells [3]. Since TMPRSS2 activation is an androgen-dependent process [3], this would represent a plausible explanation for the less pronounced susceptibility of females to SARS-CoV-2 infection in comparison to male patients [4].

Notwithstanding the above, it would be interesting to investigate if women in menopause—a life season during which an increase in total cholesterol levels is commonly found [5]—become more prone to acquire SARS-CoV-2 infection and to additionally develop, thereafter, more severe CoViD-19 forms, as the result of an enhanced virus-cholesterol-SR-B1 interaction. In this respect, it would be also worthwhile to assess whether, and to what extent, cholesterol-lowering drugs like statins [6] may interfere with SARS-CoV-2 uptake on behalf of SR-B1 and ACE-2 co-expressing cells and tissues.

Another intriguing issue within the complex host-pathogen interaction dynamics is that related to the hitherto characterized "comparative pathology models" of SARS-CoV-2 infection and CoViD-19, a number of which could faithfully recapitulate their main pathogenetic features, with special emphasis on the strategies put in place by the viral agent for host cell targeting and colonization [7].

Among these putative infection and disease models, prions appear to be, in my opinion, a particularly intriguing pathogens’ group, although we are dealing with a category of non-viral and "unconventional" agents, which are responsible for "transmissible spongiform encephalopathies" (TSE), or "prion diseases" (PD), in man and animals [8]. 

Indeed, as a veterinary and academic pathologist involved since almost 30 years in the study of TSE pathogenesis, with special reference to sheep and goat scrapie—the PD "prototype" [9],—I would like to emphasize herein that a selective binding of human prions to plasma low-density lipoproteins has been clearly documented [10]. Still noteworthy, the host "cellular prion protein" (PrPc) has been shown to be tightly associated with cholesterol-rich "lipid rafts" located in cell membranes, with such interaction(s) additionally playing an important role in the context of the conversion process of PrPc into its “pathological (or disease) counterpart” (PrPSc or PrPd) and, thereby, in prion propagation [11]. A direct connection has been also established between the level(s) of circulating blood cholesterol, on one side, and the clinico-pathological TSE progression in experimentally challenged mice, on the other [12].

Although we don’t know yet if, and to what extent, SARS-CoV-2 interacts with PrPc, the aforementioned relationships occurring between prions and plasma lipoproteins, as well as between PrPc and cell membrane lipid raft-associated cholesterol, make this topic worthy of ad hoc investigations.

While Dr Wei and coworkers should be warmly congratulated for their nice and elegant study [2], providing a lot of food for insightful thought, another recent work has identified cholesterol 25-hydroxylase (CH25H) and its enzymatic product, 25-hydroxycholesterol (25HC), as powerful inhibitors of SARS-CoV-2 replication [13]. 

In conclusion, it is my strong belief that adequate research efforts should be made in order to characterize suitable "comparative pathology models" able to recapitulate the intricate and complex interaction dynamics of SARS-CoV-2 with human cells and tissues. Albeit classified as "unconventional" (and non-viral) agents, prions—of both animal and human origin—could provide a valuable option in this direction.
